# Publisher Correction: Geographic social inequalities in information-seeking response to the COVID-19 pandemic in China: longitudinal analysis of Baidu Index

**DOI:** 10.1038/s41598-022-17295-9

**Published:** 2022-07-28

**Authors:** Zhicheng Wang, Hong Xiao, Leesa Lin, Kun Tang, Joseph M. Unger

**Affiliations:** 1 Vanke School of Public Health, Tsinghua University, No 30 Shuangqing Road, Beijing, 100084 China; 2 School of Medicine, Tsinghua University, Beijing, China; 3 China Development Research Foundation, Beijing, China; 4 Public Health Sciences Division, Fred Hutchinson Cancer Research Center, Seattle, WA 98109 USA; 5 Department of Infectious Disease Epidemiology, London School of Hygiene and Tropical Medicine, London, UK; 6 Laboratory of Data Discovery for Health (D24H), Hong Kong Science Park, Sha Tin, Hong Kong Special Administrative Region China

Correction to: *Scientific Reports* 10.1038/s41598-022-16133-2, published online 18 July 2022

In the original version of this Article, Hong Xiao was incorrectly affiliated with ‘Seattle, USA’. The correct affiliation is listed below.

 Public Health Sciences Division, Fred Hutchinson Cancer Research Center, Seattle, WA, 98109, USA

The original Article has been corrected.

